# Complete genome sequence of *Helicobacter pylori* strain NY43 harboring bacteriophage KHP30

**DOI:** 10.1128/mra.00747-26

**Published:** 2026-09-22

**Authors:** Michiko Takahashi, Hiromichi Maeda, Satoru Seo, Hiroaki Takeuchi, Jumpei Uchiyama, Shigenobu Matsuzaki

**Affiliations:** 1 Department of Surgery, Kochi Medical School, Kochi University 12888, Nankoku, Kochi, Japan; 2 Biomanufacturing Process Research Center, National Institute of Advanced Industrial Science and Technology (AIST), Tsukuba, Ibaraki, Japan; 3 Department of Medical Laboratory Sciences, Health and Science, International University of Health and Welfare Graduate School, Chiba, Japan; 4 Department of Infectious Diseases, Graduate School of Medicine, Density and Pharmaceutical Sciences, Okayama University 12997, Okayama, Japan; 5 Research Center for Intestinal Health Science, Okayama University 12997, Okayama, Japan; 6 Department of Medical Laboratory Science, Faculty of Health Sciences, Kochi Gakuen University 105224 , Kochi, Kochi, Japan; Queens College Department of Biology, Queens, New York, USA

**Keywords:** *Helicobacter pylori*, chromosome organization, bacteriophage episome

## Abstract

The *Helicobacter pylori* NY43 strain spontaneously releases the bacteriophage (phage) KHP30. Here, we report the complete genome sequence of *H. pylori* NY43. In addition to the chromosome of *H. pylori* NY43, we successfully obtained the complete sequence of the episomal prophage KHP30.

## ANNOUNCEMENT

*Helicobacter pylori* is a gram-negative bacterium responsible for certain gastric diseases (1, 2). Approximately 30% of *H. pylori* isolates harbor prophages in their genomes (3). We previously reported that the *H. pylori* NY43 strain spontaneously releases phage KHP30 (4, 5). However, the genome sequence of *H. pylori* NY43 remains undetermined. Here, we report the complete genome sequence of *H. pylori* NY43.

*H. pylori* NY43 was isolated from a gastric biopsy sample taken from a patient with a gastric ulcer at a hospital in Yamaguchi Prefecture, Japan, in 2001 (4, 5). All cultures were incubated at 37°C in the presence of 10% CO_2_. The isolated strain was cultured in BEV broth (Brucella broth [Becton Dickinson, Maryland, USA] supplemented with 10% [vol/vol] heat-inactivated equine serum [HyClone, Utah, USA] and 10 µg/mL vancomycin) and stocked at −80°C in 30% glycerol. During storage, the strain was recovered and passaged several times. For the present study, the strain was recovered on a BEV plate (1.5% agar), and a single colony was cultured in 5 mL BEV broth for 16 h. Genomic DNA was then extracted using a Maxwell RSC Genomic DNA Kit (Promega, Massachusetts, USA) according to the manufacturer’s instructions. The extracted DNA was purified using ProNex Beads (Promega), sheared to approximately 10–20 kb using a g-TUBE (Covaris, Massachusetts, USA), and used for library preparation without an additional size-selection step. A DNA library was subsequently prepared using the SMRTbell gDNA Sample Amplification Kit (PacBio, California, USA) and the SMRTbell Express Template Prep Kit 2.0 (PacBio). Sequencing was performed using PacBio Revio, generating 34,427 reads, totaling 248,007,313 bp, of which 96.8% were Q30 reads. Adapter overhang sequences were removed from the raw reads to generate subreads using SMRT Link (v11.0.0.146107) with default parameters. High-fidelity reads with quality values >20 were generated using SMRT Link and preprocessed using Filtlong (v0.2.1) (https://github.com/rrwick/Filtlong) to remove reads <1,000 bp, resulting in 29,324 clean reads. The filtered reads were then *de novo* assembled using Flye (v2.9.1-b1786) with default parameters (6). The assembled genome was assessed and autoannotated using CheckM2 (v1.0.1) and DFAST (v1.6.0), respectively (7, 8). The circular structure of the assembled contigs was also confirmed using Bandage v0.8.1 (9). Sequence comparison was performed using DiGAlign v2.0 with default parameters (10).

**Fig 1:**
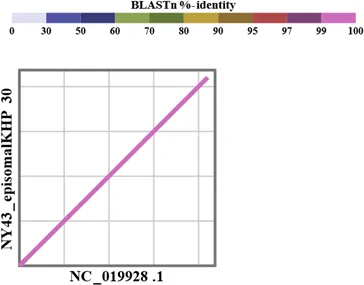
A dot plot representing the episomal sequence of KHP30 in NY43 and registered sequence of KHP30 (NC_019928.1). Each dot represents nucleotide sequence similarity identified by BLASTn.

Sequencing yielded a mean coverage of 155× and a read N_50_ value of 7,831 bp. We obtained two circular contigs: a 1,573,737-bp chromosome (G + C content 38.8%) and a 26,215-bp episomal prophage, KHP30 (G + C content 35.8%). The NY43 chromosome contains 1,527 coding sequences (CDSs), 4 rRNAs, and 36 tRNA genes, whereas the KHP30 episome contains 27 CDSs. The KHP30 episomal sequence showed >99.99% identity with the registered KHP30 sequence (accession number NC_019928.1) (Fig. 1).

## Data Availability

This genome project is indexed at DDBJ/ENA/GenBank under BioProject accession number PRJDB40718. The complete genome sequence of *H. pylori* NY43 and the episomal sequence of KHP30 have been deposited in the DDBJ/ENA/GenBank databases under accession numbers AP046640 and AP046641, respectively. The PacBio reads can be found at SRA accession number DRR919144.
